# Delayed Diagnosis of Nasal Natural Killer/T-Cell Lymphoma

**DOI:** 10.1155/2013/918132

**Published:** 2013-12-15

**Authors:** Jennifer A. Villwock, Kristin Jones, Jason Back, Parul Goyal

**Affiliations:** ^1^Department of Otolaryngology, SUNY Upstate Medical University, 750 East Adams Street, Syracuse, NY 13210, USA; ^2^Department of Pathology, SUNY Upstate Medical University, 750 East Adams Street, Syracuse, NY 13210, USA

## Abstract

Midline destructive lesions of the face have multiple possible etiologies. The majority of these cases are found to be due to an extranodal lymphoma of natural killer/T-cell-type non-Hodgkins lymphoma (NKTL). Unfortunately, diagnosis is often delayed. With variable presenting complaints, including nonspecific issues like chronic rhinosinusitis or nasal congestion, initial treatments are aimed at these presumed diagnoses. Only as the lesion progresses do overt signs of destruction occur. As with our patient, who was initially treated for presumed infection and abscess, final diagnosis often does not occur until several months, and several antibiotic courses, from initial presentation. As such, it is important for this rare entity to remain in the clinician's differential diagnosis for nasal lesion.

## 1. Introduction

Midline destructive lesions (MDLs) of the face were first reported in the late nineteenth century. These later became known as lethal midline granulomas. Other monikers such as idiopathic midline granuloma, idiopathic midline destructive disease, midline nonhealing granuloma, polymorphic reticulosis, and Stewart syndrome have also been used to describe this destructive process of the nose. Regardless of the name used, an ulcerative process, characterized by loss of both epithelium and cartilage with associated nasal crusting, occurs. Ultimately, there is loss of nasal structure and support, leading to nasal deformity [1]. After thorough workup, the majority of these cases—as in the case presented in this paper—are found to be due to an extranodal lymphoma of natural killer/T-cell lymphoma (NKTL).

Though rare, NKTL is the most common form of sinonasal lymphoma and, thus, should be included in the differential for destructive lesions of the nasal cavity or midline face. There is a strong association with Epstein-Barr virus (EBV) and other epidemiologic risk factors including Asian or Central and South American descent, male gender, and age 50 to 55 [2–4]. In the United States and other Western countries NKTL accounts for less than 2% of all non-Hodgkins lymphoma. Presentation is variable but most commonly includes nonspecific complaints of chronic rhinosinusitis, with initial treatments aimed at this presumed diagnosis. As the lesion progresses, patients may have additional complaints such as disruption of normal nasal airflow leading to epistaxis and nasal dyspnea, palatal destruction, associated orbital edema and erythema, and facial pain. Intranasal examination may reveal mild to significant crusting with extensive ulceration and destruction of the surrounding tissues [1]. Constitutional symptoms are variably present, becoming more common with disease progression.

## 2. Case Presentation

A 75-year-old Hispanic female presented with an 8-week history of a progressively enlarging nasal lesion involving the right nasal sidewall and ala, despite antibiotic treatment for presumed infection—and, later, abscess—given in multiple courses over two months. The patient denied history of trauma. In addition to obvious cosmetic deformity, she reported progressive nasal crusting, pain, intermittent right-sided epistaxis, and nasal dyspnea. She was admitted to the Otolaryngology service for intravenous antibiotics for what was thought to be a nasal abscess.

Physical examination revealed an exophytic mass involving the entirety of the right nasal ala with extension onto the nasal sidewall. The skin overlying the lesion was erythematous with central crusting suggestive of ulceration. The lesion was firm and fixed to the surrounding soft tissue and underlying alar cartilage. Nasal endoscopy revealed extensive right-sided nasal crusting and friable tissue along the undersurface of the nasal ala and nasal sidewall. Fine needle aspiration was attempted but nondiagnostic. A biopsy of the right nasal alar lesion was obtained and sent for formal histopathologic examination.

**Figure 1:**
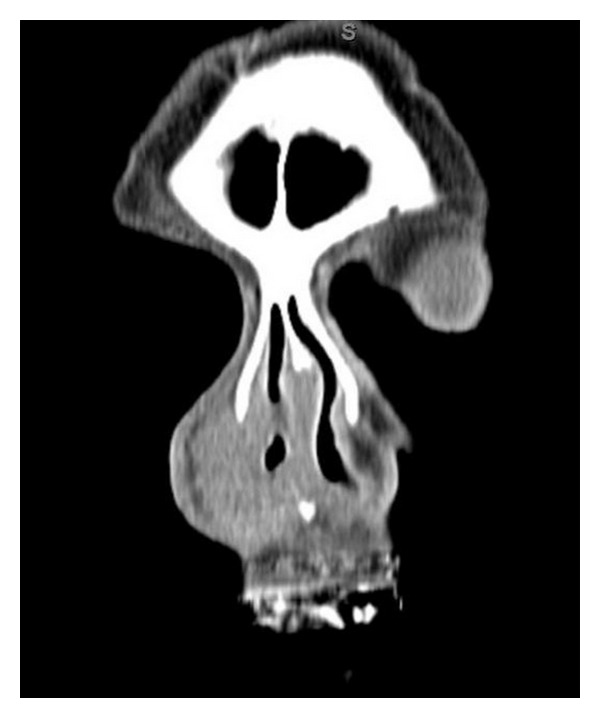
Coronal contrast enhanced CT scan showing large subcutaneous soft tissue density lesion of the nasal ala.

**Figure 2:**
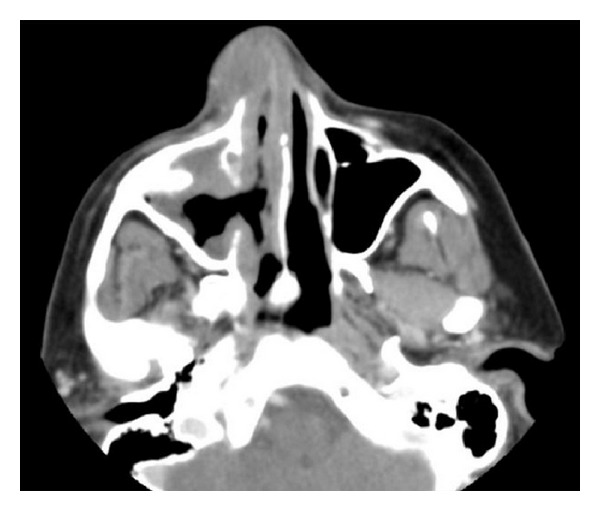
Axial contrast enhanced CT scan redemonstrating subcutaneous nasal lesion.

**Figure 3:**
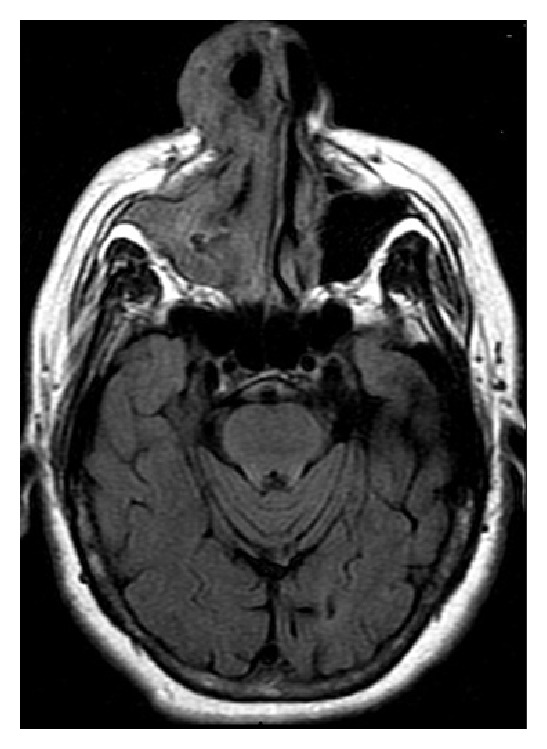
Axial MRI T2 FLAIR image of the nasal lesion with relatively homogeneous, low intensity signal.

Maxillofacial computed tomography (CT) revealed an extensive nonenhancing submucosal lesion of soft tissue density involving the entirety of the right nasal ala, with associated distortion of the columella due to mass effect. The lesion appeared to be in a subcutaneous plane. The mucosal surface also appeared to be preserved. There was an incidental finding of benign-appearing mucosal thickening seen within the right maxillary and ethmoid sinuses (Figures 1, 2, and 3).

**Figure 4:**
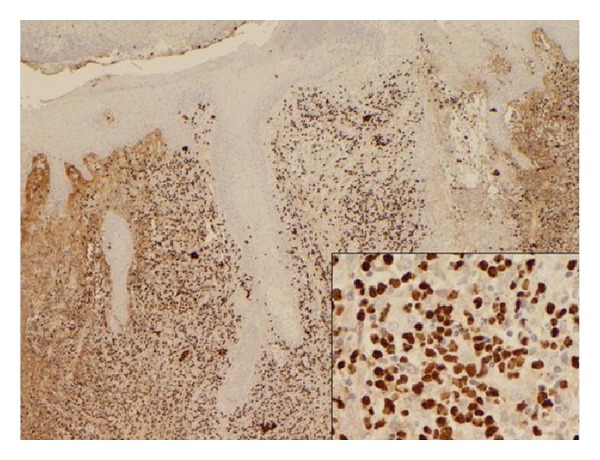
Epstein-Barr virus encoded ribonucleic acid (EBER) in situ hybridization showed strong nuclear staining in the majority of cells. Inset is area of higher magnification.

Histologic findings demonstrated pseudoepitheliomatous hyperplasia and ulceration overlying a diffuse mixed inflammatory infiltrate consisting predominantly of histiocytes and lymphocytes. Epstein Barr virus encoded ribonucleic acid (EBER) in situ hybridization showed strong nuclear staining in the majority of cells (Figure 4).

## 3. Discussion

Much of the dilemma with midline destructive lesions of the face is in diagnosis. However, establishing the diagnosis quickly is imperative so that proper therapy can be initiated without delay. As seen with our patient who was initially treated with multiple courses of antibiotics, the diagnostic approach tends to be difficult as symptoms are nonspecific and the differential diagnosis is extensive, including infectious, autoimmune, neoplastic, and inflammatory etiologies. The initial laboratory workup includes a complete blood count with differential. Further testing for specific disease and autoimmune entities may include classic antineutrophil cytoplasmic antibodies (cANCAs), perinuclear antineutrophil antibodies (pANCAs), proteinase 3 (PR3), myeloperoidase (MPO), angiotensin converting enzyme (ACE), rapid plasma reagin (RPR), anti-Sjogren A and B antibodies, rheumatoid factor (RF), coccidioidomycosis, and human immunodeficiency virus (HIV) testing [3]. Imaging usually reveals an infiltrative soft tissue mass that partially obliterates or obstructs the nasal passages (Figure 1). Ultimately, tissue biopsy is required for diagnosis. Fine needle aspiration (FNA) can be attempted but may be nondiagnostic due to the presence of extensive necrosis. Similarly, even biopsy specimens may be difficult to interpret requiring multiple biopsies [1].

Classic histologic features include angiocentric and angiodestructive growth with zonal necrosis. Immunohistochemistry (IHC) can then differentiate NKTL from other lymphoproliferative malignancies. The expression of CD3 and CD56 differentiates NK/T-cell lesions from other T-cell lymphomas [5]; in our specimen these markers were strongly positive. Interestingly, unlike normal NK cells, they are CD16 and CD57 negative. Epstein Barr virus encoded ribonucleic acid (EBER) in situ hybridization is used to illustrate the presence of EBV in samples, further highlighting the association between NKTL and EBV [1, 5]. Coexpression of the above markers and EBER positivity strongly support a diagnosis of extranodal NKTL, nasal type. Interestingly, our patient also expressed CD4, which is an uncommon feature.

The radiologic findings of extensive soft tissue and sinonasal involvement yield a differential diagnosis that includes granulomatosis with polyangiitis (formerly Wegener's granulomatosis), other granulomatous infections, other non-Hodgkins lymphoma, adenoid cystic carcinoma, olfactory neuroblastoma, melanoma, and squamous cell carcinoma [6, 7]. Overall, the imaging characteristics of NKTL are relatively nonspecific. However, there are features that can suggest the diagnosis. Mild to moderate heterogenous enhancement of the involved soft-tissue on both CT and MRI imaging is generally present. Bony erosion is reportedly present in 40 to 78% of patients with the most common sites being the medial maxillary wall, the nasal septum, and the lamina papyracea. Despite marked soft-tissue involvement of the midface, the anterior maxillary sinus wall is rarely involved. Additionally, the bony involvement of NKTL is less severe than other destructive entities such as sinonasal squamous cell carcinoma. On MRI, tumoral tissue appears isointense on T1-weighted images and hyperintense on T2-weighted sequences. T2-weighted images help differentiate retained secretions (markedly hyperintense) from tumor tissue which is heterogeneously hyperintense [6]. In the case presented, the lesion did not appear to arise from the cutaneous and mucosal surfaces, making the diagnosis of an epithelial neoplasm less likely. In a patient with a subcutaneous nasal soft tissue lesion, this entity should be considered.

The clinical course is aggressive with five year survival rates ranging from 38 to 45 percent [1, 8, 9]. The EBV viral load, the degree of angiocentricity and angioinvasion, stage, and time to diagnosis are the main prognostic variables [10]. Staging is currently per the Ann Arbor staging system for lymphoma; however, recent studies have demonstrated local invasiveness as an independent risk factor for poor prognosis [10, 11]. Based on this data, a new staging system has been proposed in which the disease is staged as either limited or extensive.

The current mainstay of treatment is radiotherapy for all patients with localized disease with the optional addition of an anthracycline-based chemotherapy, such as CHOP (cyclophosphamide, doxorubicin, vincristine, and prednisolone) or CHOP-like regimen. Unfortunately, due to the relative rarity of NKTL, there is a lack of consensus on radiation field, optimal dose, and chemotherapy regimen [10, 12]. The local recurrence rate is reported to be as high as 50% with a high rate of distant metastases within two years of treatment which is thought to be due to a multidrug resistance mechanism [10, 11]. Given this high recurrence rate with radiotherapy and chemotherapy, high dose chemotherapy and autologous hematopoietic stem cell transplantation are currently being investigated as a treatment for NKTL. Preliminary studies show promise for this therapy providing a survival benefit when transplant is performed during the first complete remission [13].

## 4. Conclusion

MDLs are most often attributed to NKTL. As mentioned in the literature, and demonstrated by our patient, there is often a delay in diagnosis. This is largely due to the lack of specificity in presenting symptoms and the overall diagnostic difficulty MDL's present. A thorough evaluation, including appropriate imaging, laboratory studies, and biopsy specimens, is warranted for midline lesions that do not respond to conventional medical therapy. IHC is key in establishing the diagnosis. Prompt initiation of treatment in the form of radiotherapy and/or chemotherapy is critical for optimization of patient outcome.
